# A novel mechanism for treating acute lung injury with ligustilide: elucidation via network pharmacology and in vitro validation

**DOI:** 10.1007/s11418-026-02011-y

**Published:** 2026-03-21

**Authors:** Yi Yu, Zhennan Wang, Chentong Pan, Keyao Zhu, Liying Xu

**Affiliations:** 1 The First Clinical Medical College, Zhejiang Chinese Medical University, Zhejiang, 310,053 China; 2 Xianju County Hospital of Traditional Chinese Medicine, Taizhou, Zhejiang 317,300 China; 3 Department of Medical Administration, The First Affiliated Hospital of Zhejiang Chinese Medical University (Zhejiang Provincial Hospital of Chinese Medicine), 54 Youdian Road, Hangzhou, Zhejiang 310,006 China

**Keywords:** Ligustilide, Network pharmacology, Autophagy, Acute lung injury, PI3K/Akt/mTOR signaling pathway, Interleukin 17A

## Abstract

Ligustilide inhibits the release of multiple inflammatory factors and alleviates acute lung injury (ALI), although its underlying mechanisms require further investigation. This study combined network pharmacology and in vitro experiments to systematically elucidate the therapeutic mechanism of ligustilide against lipopolysaccharide (LPS)-induced ALI. Potential targets of ligustilide and ALI-related genes were screened from public databases, identifying 227 overlapping targets. Protein-protein interaction (PPI) network analysis revealed PIK3R1 as core targets. KEGG pathway enrichment analysis highlighted the PI3K/Akt/mTOR signaling axis as one of the most significantly enriched pathway (*P* < 0.001). Molecular docking confirmed a strong binding affinity between ligustilide and the PIK3R1 protein. In vitro, LPS exposure (10 µg/ml, 48 h) suppressed autophagy in HPAEpiCs, evidenced by reduced Beclin-1 and LC3 protein levels and a decrease in autophagic vesicles, while concurrently activating the PI3K/Akt/mTOR signaling pathway (*P* < 0.01) and elevating the levels of IL-17 A, IL-6, and TNF-α. Treatment with ligustilide (25 µg/ml) significantly reversed these effects by modulating autophagy-related markers and suppressing the release of these inflammatory cytokines (*P* < 0.05). Furthermore, co-treatment with anti-IL-17 A neutralizing antibodies amplified these protective effects through enhanced inhibition of the PI3K/Akt/mTOR pathway (*P* < 0.01). These findings suggest that ligustilide alleviates ALI by modulating autophagy-related markers and inhibiting inflammatory responses via the IL-17 A-mediated PI3K/Akt/mTOR signaling pathway. The integration of network pharmacology with experimental validation provides a robust mechanistic framework for understanding ligustilide’s therapeutic potential against ALI.

**Graphical abstract:**

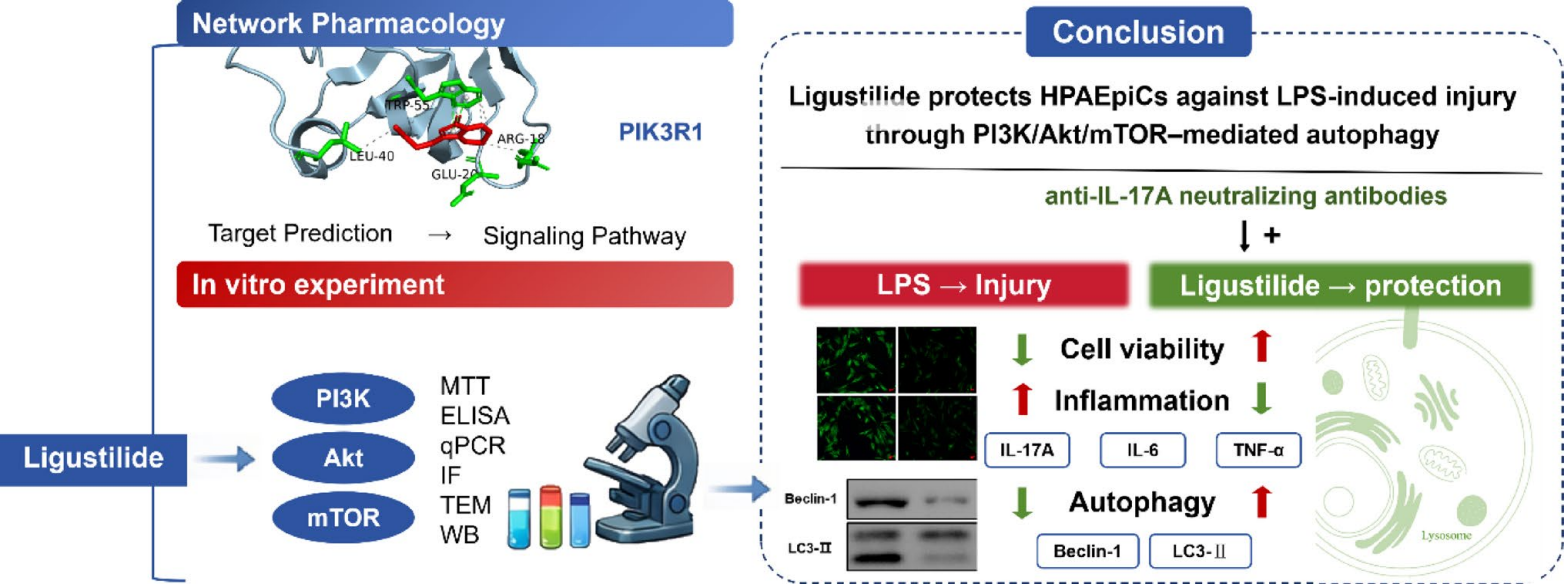

**Supplementary Information:**

The online version contains supplementary material available at 10.1007/s11418-026-02011-y.

## Introduction

Acute lung injury (ALI) is characterized by an inflammatory onset, manifesting as uncontrolled oxidative damage, inflammatory response, and autophagic cell death [1]. Subsequently, inflammatory cytokines and chemokines mediate mutual amplification and perpetuation of lung injury, triggering an inflammatory cascade that may progress to acute respiratory distress syndrome (ARDS) [2, 3]. The mortality rate of severe ARDS is as high as 45%, and during the initial outbreak of the coronavirus disease 2019 (COVID-19) pandemic, the mortality rate even reached a staggering 67–85% [4, 5]. Currently, providing respiratory support and improving patient oxygenation are the primary treatments for ALI, and there are no clinically approved drugs that significantly reduce ALI/ARDS mortality [1]. Therefore, a thorough understanding of the etiology of ALI and early intervention are crucial for slowing the progression of primary diseases such as pneumonia and reducing morbidity.

In contrast to the classical dichotomy of apoptosis and necrosis, autophagy has become a research focus in the pathogenesis of ALI. Previous studies have found that autophagy alleviates uncontrolled inflammation in ALI mice through pathways such as NF-κB and Akt-TSC2-mTOR [6–8]. Inhibiting autphagy can reduce lung injury and pulmonary edema, thereby exerting a lung-protective effect [9]. Inadequate autophagy is one of the pathogenic mechanisms in lipopolysaccharide (LPS)-induced ALI.

Ligustilide, the main active phthalide component of *Angelica sinensis*, possesses significant anti-inflammatory and organ-protective effects. It has been reported to exert protective effects against LPS-induced ALI [10]. In addition, previous research has shown that ligustilide can inhibit the release of inflammatory factors including IL-17, thereby alleviating pulmonary edema, hemorrhage, inflammatory cell infiltration, and alveolar structural damage [11]. Previous studies have demonstrated through in vitro experiments that IL-17 A can activate the PI3K/Akt/mTOR signaling pathway, leading to decreased expression of the autophagy markers Beclin-1 and the LC3-Ⅱ/LC3-Ⅰ ratio [12]. This signaling pathway has also been shown to regulate autophagy in LPS-induced ALI, thereby influencing inflammatory responses [13]. Therefore, we hypothesize that ligustilide blocks the IL-17 A-mediated PI3K/Akt/mTOR signaling pathway, enhances autophagy activity, reduces LPS-induced ALI, and acts as a lung-protective factor.

Network pharmacology analysis can efficiently identify key targets and potential pathways, construct a “drug-target-pathway” network, and provide a more objective and comprehensive research perspective [14]. This study, based on network pharmacology methods combined with molecular docking and in vitro cell experiments, systematically investigates the potential targets and pathways of ligustilide in the treatment of ALI, providing a reliable theoretical foundation for the lung-protective effects of ligustilide and the development of related drugs.

## Materials and methods

### Network pharmacology analysis

**Fig. 1:**
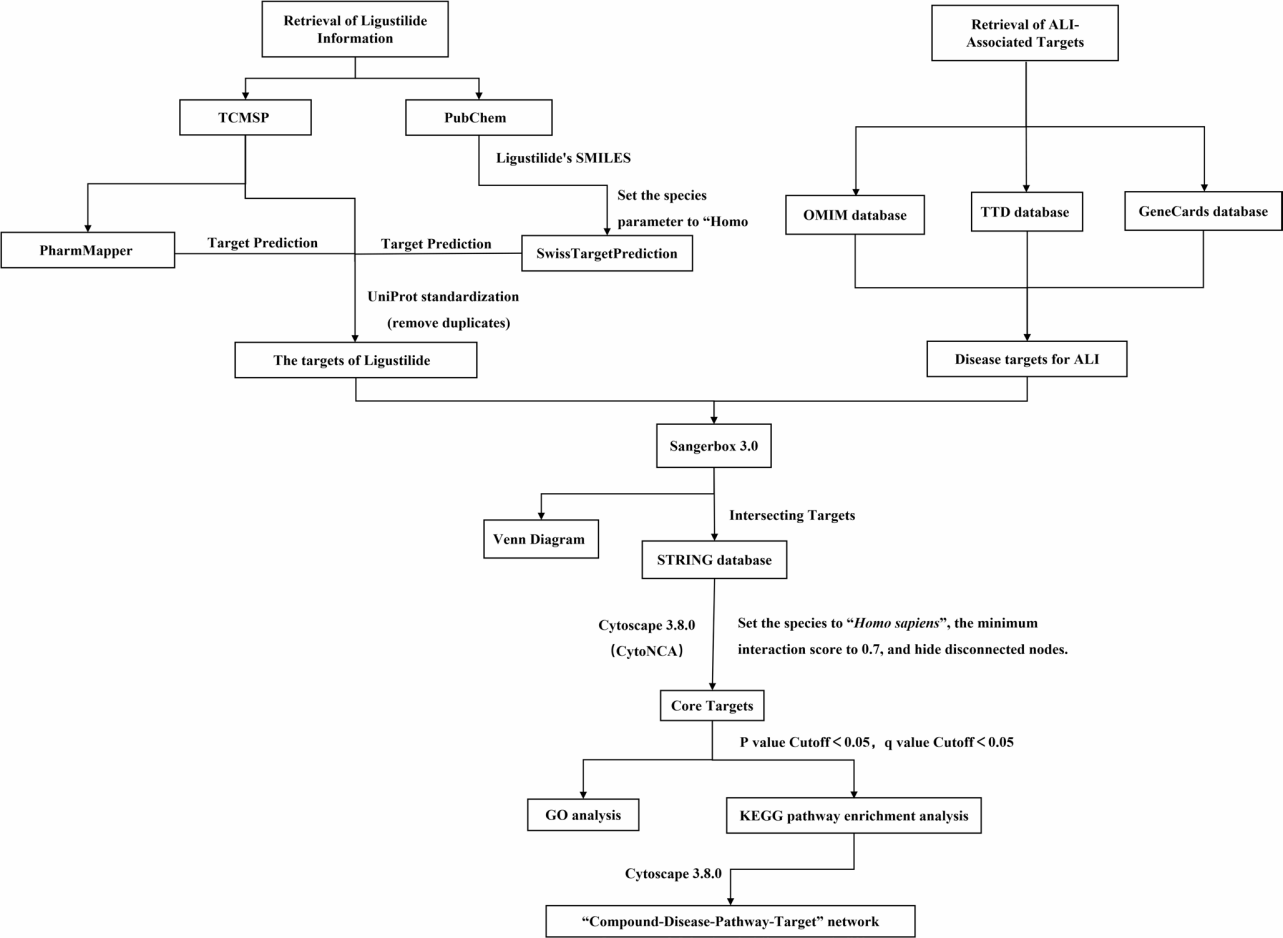
Schematic workflow of the network pharmacology analysis of ligustilide in ALI. The pipeline includes target collection, target intersection, PPI analysis, GO/KEGG enrichment, network construction, and molecular docking

An overview of the network pharmacology workflow is shown in Fig. 1. Key screening criteria and parameter settings for each step are specified in Sect. Obtaining targets associated with Ligustilide and ALI– Molecular Docking Validation.

#### Obtaining targets associated with ligustilide and ALI

The PubChem database (https://pubchem.ncbi.nlm.nih.gov/) was utilized to obtain the SMILES name of “Ligustilide” [15], which was then imported into the SwissTargetPrediction database (http://swisstargetprediction.ch/) to obtain Ligustilide’s corresponding targets (probability > 0) with species parameter set to “Homo sapiens” [16]. The chemical structure file and potential targets of ligustilide were retrieved from the TCMSP database (http://tcmspw.com/tcmsp.php) using “Ligustilide” as the search term [17]. Concurrently, the structure file was uploaded to PharmMapper (https://lilab-ecust.cn/pharmmapper/submitfile.html) for complementary target prediction. The obtained Uniprot IDs were standardized using the Uniprot database (https://www.uniprot.org/). All predicted targets were aggregated and deduplicated to establish the comprehensive ligustilide-related target dataset. To minimize potential false-negative results, a relatively inclusive probability threshold was applied at the initial target prediction stage, allowing comprehensive coverage of potential ligustilide-associated targets for subsequent network analysis.

#### Obtaining targets associated with ALI

Disease-associated targets were systematically collected from three repositories: OMIM (https://www.omim.org/), GenCards (https://www.genecards.org/) (Relevance score exceeding the median value) [18], and TTD (https://db.idrblab.net/ttd/) using “Acute Lung Injury” as the query term. ALI-related targets from all databases were consolidated and deduplicated to generate the dataset. This strategy ensured the inclusion of disease-relevant targets while reducing potential bias from individual databases.

#### Protein-Protein interaction (PPI) network construction

The ligustilide-related targets and ALI-related targets were imported into the bioinformatics visualization tools of Sangerbox 3.0 (http://sangerbox.com/home.html) to identify overlapping targets. These targets were submitted to the STRING database (https://cn.string-db.org/) with parameters: “Homo sapiens” species, minimum interaction score ≥ 0.7, and hidden disconnected nodes [19]. A high-confidence interaction threshold was selected to enhance the reliability of the constructed PPI network. The resultant PPI network was imported into Cytoscape 3.8.0 for topological analysis. Core targets were identified using CytoNCA plugin through betweenness Degree Centrality (DC) and Betweenness Centrality (BC) (exceeding twice the median values) [20]. Nodes meeting these criteria were considered to play central roles in the network and were therefore defined as core targets for subsequent functional analysis.

#### Gene ontology (GO) and Kyoto encyclopedia of genes and genomes (KEGG) pathway enrichment analysis

GO functional annotation (biological process (BP), molecular function (MF), cellular component (CC)) and KEGG pathway enrichment were performed using R packages “clusterProfiler” and “enrichplot”. Significant terms were filtered (adjusted *P* < 0.05, q < 0.05) with the top 30 KEGG pathways visualized as bubble plots. A “Compound-Disease-Pathway-Target” network was reconstructed in Cytoscape 3.8.0 for integrative visualization.

#### Molecular docking validation

The 3D structure of ligustilide (SDF format) was downloaded from PubChem and energy-minimized in Chem3D. The crystal structure of PIK3R1 (PDB ID: 4L23) was obtained from RCSB PDB (https://www.rcsb.org/). AutoDock Vina 1.5.7 performed molecular docking after protein preparation (water removal, hydrogen addition, charge calculation). Binding conformations were visualized using PyMOL with ligand-receptor interactions analyzed.

### Validation by in vitro cell experiments

#### Culture and passaging of HPAEpiCs

HPAEpiCs (batch No. HUM-iCell-a002) were purchased from iCell Bioscience Inc. (Shanghai, China). They were maintained at 37 °C in a humidified incubator with 5% CO₂. For passaging, the medium was removed and cells were gently rinsed twice with PBS, followed by brief trypsinization for 1 min. Detachment was monitored under a microscope, and trypsinization was terminated when 90% of cells became rounded and started to detach by adding complete medium. Cells were gently resuspended, collected by centrifugation (1000 rpm, 5 min), resuspended in fresh complete medium, and subcultured into new T25 flasks as required. Passage numbers were recorded throughout the study.

#### Establishment of ALI cell model

HPAEpiCs were randomly divided into four groups and 10 µg/ml LPS (batch No.039M4004V, sigma, USA) was applied for 0 h, 4 h, 24 h and 48 h respectively to determine the optimal duration of LPS action based on cellular activity and inflammatory factor release.

##### Detection of cell activity by MTT method

MTT kit was purchased from BBI Life Sciences Corporation (Shanghai, China). HPAEpiCs in the logarithmic growth phase were seeded into 96-well plates. After the indicated treatments, 10 µL of MTT solution was added to each well and incubated at 37 °C. Cell viability was assessed by measuring the absorbance at 562 nm using a microplate reader (CMax Plus, Molecular Devices, USA). Five replicate wells were included for each group.

##### ELISA was used to detect changes in the levels of IL-6 and TNF-α in the supernatant of each group of HPAEpiC cultures

The cell culture supernatant was extracted, centrifuged for 20 min (2000–3000 rpm) using Micro17R cryogenic high-speed centrifuge (Thermo Scientific, USA), and then placed in a container and kept at -80 °C. Following the manufacturer’s instructions, ELISA kits (Jiangsu Enzyme Free Industry Co. Ltd., China) were used to measure the levels of IL-6 and TNF-α. Six times were added to the test.

#### Ligustilide concentration selection

HPAEpiCs were split into 11 groups: a blank control (NC) and 10 experimental groups that underwent ligustilide (HPLC ≥ 98%, Shanghai Yuanye Bio-Technology Co., Ltd., China, batch No. B2042) interventions at various doses (0.78, 1.56, 3.125, 6.25, 12.5, 25, 50, 100, 200, and 500 µg/ml). A ligustilide stock solution (50 mg/ml; 1 mg ligustilide dissolved in 20 µL DMSO) was prepared and subsequently diluted with complete culture medium to the desired working concentrations. Changes in cell activity were monitored using the MTT method previously described. Cells were incubated with ligustilide for 24 h, after which cell viability was assessed using the MTT assay as previously described. Data were collected from five technical replicate wells per group.

#### Cell viability assay under LPS and ligustilide co-treatment

To further assess the cytoprotective effect of ligustilide under LPS-induced injury, we performed an additional MTT assay under co-treatment conditions. HPAEpiCs were seeded in 96-well plates and allowed to adhere overnight. Cells were assigned to the following groups: (i) Control; (ii) LPS only (10 µg/ml); (iii) LPS + ligustilide co-treatment, where ligustilide (25 µg/ml) and LPS (10 µg/ml) were added simultaneously; and (iv) ligustilide pretreatment, where cells were pre-incubated with ligustilide (25 µg/ml) for 4 h prior to LPS (10 µg/ml) exposure, with ligustilide maintained during LPS stimulation. Cell viability was assessed using the MTT protocol described above. Six replicate wells were included for each group.

#### Cell grouping and treatment

Well-grown HPAEpiC were injected in 12-well plates at a concentration of 1 × 10^5 cells/ml after being digested with 0.25% trypsin (batch No.SH30042.01, Cyclone, USA), and then they were randomly separated into four groups. The marks are as follows for the purpose of records: (i) Control group; (ii) model group (LPS); (iii) ligustilide group (LPS + ligustilide); and (iv) Anti-IL-17 A neutralizing antibody group (LPS + ligustilide + Anti-IL-17 A neutralizing antibody).

Ligustilide (25 µg/ml) and anti-IL-17 A neutralizing antibodies (1:500, batch No.DF6127, Affinity Antibodies, USA) were added at the ideal concentration and incubated for 4 h after HPAEpiC expanded to 60–70% density. Pretreatment therefore allows sufficient time to activate biological pathways before the injury is initiated. 10 µg/ml of LPS was added after incubation, and cells were harvested after 24 h. 1 ml/well of Trizol reagent (batch No. B511311, Bioengineering Shanghai Co., Ltd., China) was added to each well to extract total RNA and protein. After the best action time for usage, the cell supernatant was collected.

#### Determination of IL-17 A, IL-6 and TNF-α in the supernatant of each group of cells by ELISA.

Using the previously stated ELISA technique, the experiment was carried out 10 times.

#### Real-Time PCR method to determine the mRNA expression levels of Beclin-1 and MAP1LC3B (LC3B) in each group of cells

RNA was isolated from the cells by Trizol one-step extraction, agarose gel electrophoresis was performed to identify its purity by EPS300 electrophoresis instrument (Shanghai, China Tanon Technology Co., Ltd.), nucleic acid concentration was measured by mixing with RNA-free deionized water, the amount of nucleic acid was calculated, and cDNA was synthesized by reverse transcription (batch No. CW2569, Kangwei Century Biotechnology Co.). Reaction conditions: 42 °C, 15 min; 85 °C, 5 min.

According to the primer design principle, the DNA and mRNA of the test genes, Beclin-1 and LC3B, as well as the sequence of the internal reference gene, GAPDH, were located in the PUBMED database. Primer Premier 5.0 was used to construct the upstream and downstream primers (Table 1). Then perform real-time fluorescence quantitative PCR reactions (CFX Connect, BIO-RAD, USA). Conditions: 95 °C, 10 min denaturation; 95 °C, 15 s; 60 °C, 60 s; 40 cycles. All experiments were performed in triplicate.

**Table 1:** Primer sequences for PCR

Gene	Forward Primer	Reverse Primer
Human Beclin-1	5’-CTGGTAGAAGATAAAACCCGGTG-3’	5’-AGGTAGAGCGTGGACTATCCG-3’
Human LC3B	5’-GGTTTCCCGTCACCAATTTTCC-3’	5’-TGTGGTTTCCAACGTAGAGGA-3’
Human GAPDH	5’-GGAGCGAGATCCCTCCAAAAT-3’	5’-GGCTGTTGTCATACTTCTCATGG-3’

#### Immunofluorescence detection of Beclin-1 and LC3 expression in various groups of cells

The plates were seeded at 60–70% cell density, and the drugs were individually added after walling, fixed with 4% paraformaldehyde (PFA) for 10 min, and rinsed 3 times with pH 7.2–7.4 phosphate buffer (PBS) (Cyclone, USA) for 5 min each time; 1–2 ml of 0.5% Triton X-100 was added and treated for 2 min to permeabilize the cell membrane, rinsed in the same way, and closed with an appropriate amount of 3% BSA blocking solution for 0.5 h and incubated the primary antibody. Repeated the rinsing step; incubated the secondary antibody and kept all subsequent steps away from light. Rinse twice, stained with DAPI, rinse once, and added 1 ml of PBS to keep wet. Added an appropriate amount of blocking agent Mounting media, and capillary aspirated nail polish to seal the film; observed and photographed under the inverted fluorescent microscope (Ts2-FC, Nikon Corporation, Japan). Fetal Bovine Serum (batch No.11011 − 8615) was purchased from Zhejiang Tianhang Biotechnology Co. (China) and Goat anti-rabbit IgG H&L (Alexa Fluor^®^ 488) (batch No. ab150077) from Abcam Plc. (UK). All experiments were independently repeated three times.

#### Transmission electron microscope observation of autophagy in each group of HPAEpiC

Collected cells were fixed in 2.5% glutaraldehyde solution for 4 h, rinsed 4 times in PBS for 15 min each, fixed in 1% osmium acid solution for 1–2 h, rinsed 3 times; dehydration in a gradient of ethanol solution (50%, 70%, 80%, 90%, 95% and 100%) for 15 min in each gradient except 100% treatment for 20 min, and finally treated with pure acetone for 20 min. Samples were treated with an embedding agent acetone mixture, (V/V = 1/1) ratio for 1 h, (V/V = 3/1) for 3 h, overnight at room temperature; placed in 0.5 ml dry centrifuge tubes, heated and polymerized overnight at 70 °C; trimmed and sectioned (50–70 nm) on a Leica EM UC 7 ultrathin sectioning instrument; plastic embedding moulds, 50% Uranium acetate 50% ethanol saturated solution 100 µL staining (15 min–1 h), double-distilled water rinse with 100 µL of lead citrate for 15 min; electron microscopy (H-7650, Hitachi Limited, Japan) photographed. Data represent three independent experiments.

#### Western blot assay for the protein expression levels of Beclin-1, LC3, Akt, p-Akt, mTOR and p-mTOR in cells

Discarded supernatant. Cells were washed twice with PBS before centrifuging at 1000 rpm for 5 min, the supernatant removed, lysed on ice with RIPA lysis solution for 30 min, centrifuged at 12,000 g, 4 °C for 5 min, supernatant taken as protein sample; BCA method (batch No.pc0020, Solarbio, Beijing) was used to determine the total protein concentration of the samples; 10% separation gel and 5% concentration gel were configured according to the instructions, injected 1× Tris-Gly’s electrophoresis buffer, add the sample and pre-stained protein marker (batch No.PR1910, Solarbio, Beijing), and keep the gel surface in equilibrium with 1× SDS loading buffer. The initial voltage was 80 V, and the protein sample was lifted to 120 V after entering the separation gel for electrophoresis. After transferring the membrane well (VE186, Tanon, China) marked, the membrane was washed 10 min x 3 times with TBST. Conditions: Regulated current 200 mA, 120 min.

The PVDF membrane (GE Healthcare Life, USA) was closed with 5% skimmed milk powder in a blocking solution and shaken for 1.5–2 h. The membrane is washed as above; the membrane was incubated overnight at 4 °C in an incubator containing primary antibody dilution (according to the instructions) and shaken at room temperature; the next day the membrane was removed and shaken at room temperature for 30 min, the primary antibody (Anti-rabbit IgG, CST, USA) was aspirated and discarded and the membrane was washed; the secondary antibody (HRP-linked Antibody, CST, USA) was diluted in blocking solution and shaken at room temperature for 1–2 h. After the reaction, the secondary antibody was recovered and the membrane was washed. The PVDF membranes were exposed to the ECL reagent for 3 min; images were taken using chemi capture software, chemical light, and automatic exposure. All assays were independently repeated three times. Beclin-1, LC3, Akt, p-Akt, mTOR, p-mTOR and GAPDH Antibodies were purchased from Affinity Biosciences Pty Ltd. (USA). The LC3 antibody recognizes both LC3-I and LC3-II forms, as confirmed by the presence of two bands in WB.

####  Statistical analysis

Statistical analyses were performed using SPSS 16.0. Data are expressed as mean ± SD. Comparisons among multiple groups were conducted using one-way ANOVA followed by Dunnett’s post hoc test. A value of *P* < 0.05 was considered statistically significant.

## Results

### Network pharmacology analysis

#### Pharmacological data of ligustilide

**Fig. 2:**
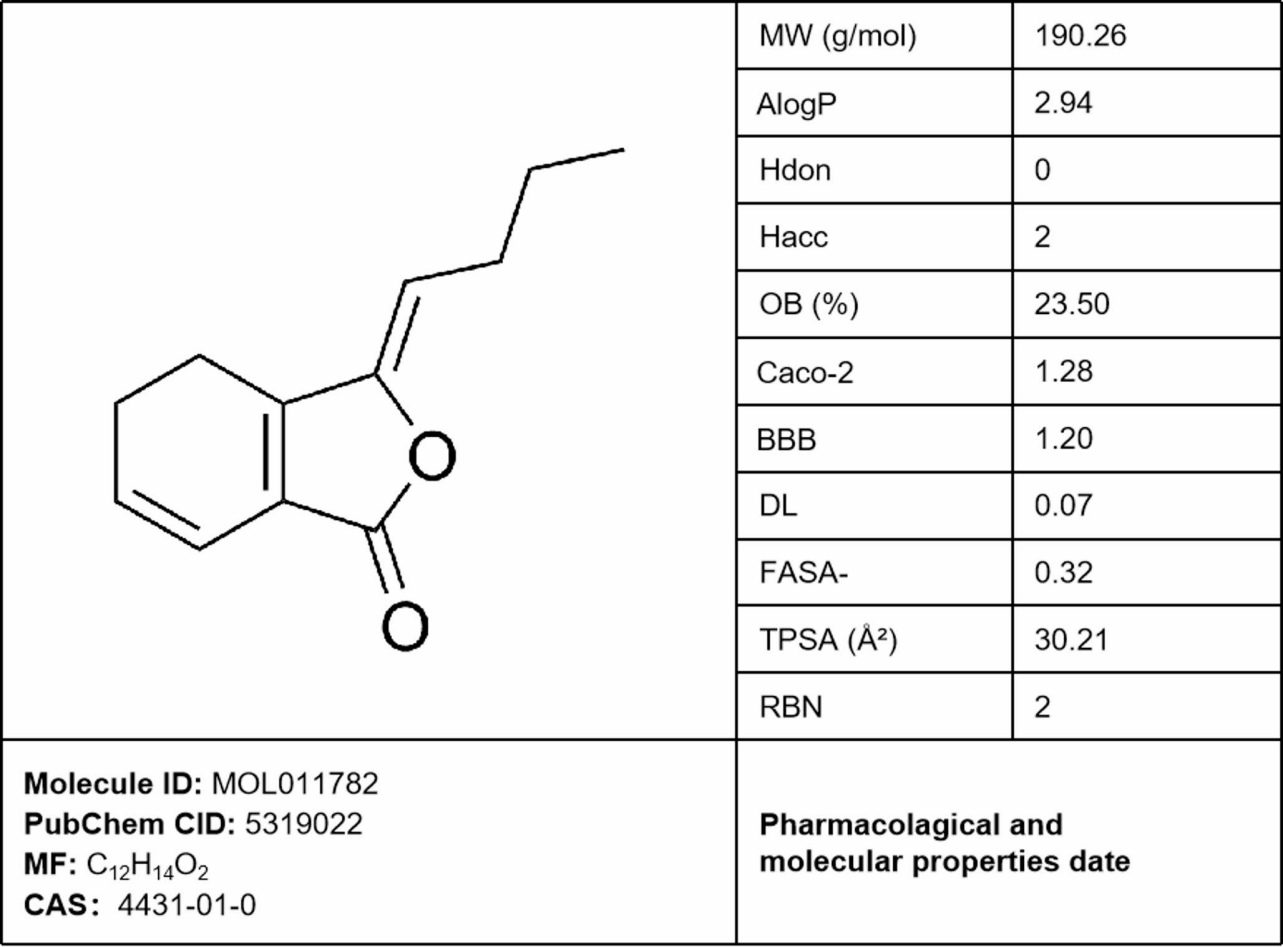
Basic pharmacological properties of ligustilide

Based on the pharmacological data presented in Fig. 2, ligustilide exhibits a moderate molecular weight (190.26 Da) and optimal lipophilicity (AlogP = 2.94), aligning with Lipinski’s Rule of Five criteria for oral bioavailability. The molecule possesses zero hydrogen bond donors (Hdon = 0), only two hydrogen bond acceptors (Hacc = 2), and a notably low topological polar surface area (TPSA = 30.21 Å²), collectively indicating minimal polarity-related barriers to passive membrane diffusion. This is further supported by a high predicted Caco-2 cell permeability value (1.28), suggesting efficient intestinal absorption. The compound’s predicted oral bioavailability (OB = 23.5%) falls within an acceptable range for further development. Crucially, the significant positive blood-brain barrier penetration coefficient (BBB = 1.20) strongly suggests a high propensity for CNS distribution. While the drug-likeness score (DL = 0.07) is suboptimal, potentially reflecting structural uniqueness, low molecular flexibility (RBN = 2) support overall favorable physicochemical properties. The fraction of sp3 carbon atoms (FASA-=0.32) indicates reasonable three-dimensional complexity. Collectively, these parameters suggest a molecule with good membrane permeability, moderate oral bioavailability potential, and a high likelihood of reaching CNS targets, warranting further investigation despite the lower DL score. Although the DL value of ligustilide does not meet the conventional criterion of 0.18, extensive research has demonstrated its pharmacological activity in treating respiratory diseases [10, 21]– [22]. Subsequent in vitro experimental results further substantiate this conclusion. This also suggests that researchers should not overly rely on specific numerical thresholds for screening active ingredients when conducting network pharmacology studies.

#### Target prediction of ligustilide in treating ALI

**Fig. 3:**
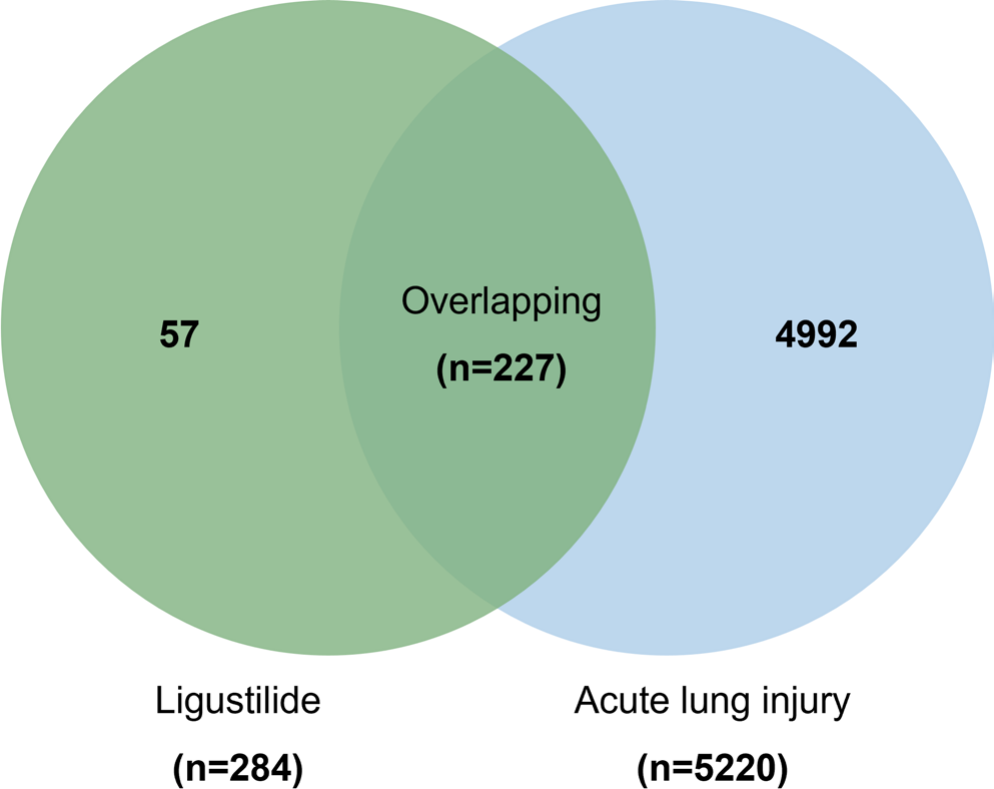
Identification of overlapping targets between ligustilide and ALI

To investigate the potential molecular targets of ligustilide in treating ALI, network pharmacology analysis was performed. Initially, 101, 2, and 201 genes associated with ligustilide were identified using the SwissTargetPrediction, TCMSP, and PharmMapper databases, respectively. After removing duplicates, 284 unique ligustilide-related targets were obtained. ALI-related genes were retrieved from the Homo-OMIM, GenCards, and TTD databases, yielding 5,220 genes post-deduplication. A Venny diagram generated via the Sangerbox 3.0 platform revealed 227 overlapping targets between the compound and disease (Fig. 3).

#### PPI network construction

PPI network comprising 227 nodes and 865 edges was constructed to visualize ligustilide’s effects on ALI (Supplementary Figure S1), with an average node degree of 7.62. Key targets mediating ligustilide’s anti-ALI activity are illustrated in Supplementary Figure S2. Using the CytoNCA plugin, 25 core targets were identified through sequential filtering based on Degree Centrality (DC) and Betweenness Centrality (BC) values exceeding twice the median. The top 10 core targets were identified as Signal Transducer and Activator of Transcription 3 (STAT3), Epidermal Growth Factor Receptor (EGFR), Heat Shock Protein 90 Alpha Family Class A Member 1 (HSP90AA1), SRC Proto-Oncogene, Non-Receptor Tyrosine Kinase (SRC), Heat Shock Protein 90 Alpha Family Class B Member 1 (HSP90AB1), Phosphoinositide-3-Kinase Regulatory Subunit 1 (PIK3R1), Estrogen Receptor 1 (ESR1), Janus Kinase 2 (JAK2), Matrix Metallopeptidase 9 (MMP9), Caspase 3 (CASP3) (Table 2).

**Table 2:** Quantitative statistical tables for core nodes

Gene Symbol	Degree	Betweenness	Closeness
STAT3	50	4714.876441	0.157421289
EGFR	45	5096.882076	0.160060976
HSP90AA1	42	3871.124181	0.156133829
SRC	41	1626.916758	0.153061224
HSP90AB1	30	996.6620571	0.150537634
PIK3R1	30	793.7578648	0.149892934
ESR1	30	2180.964973	0.155670867
JAK2	26	700.0067663	0.14978602
MMP9	25	2377.009745	0.154411765
CASP3	25	1658.406117	0.153284672

#### GO enrichment analysis

**Fig. 4:**
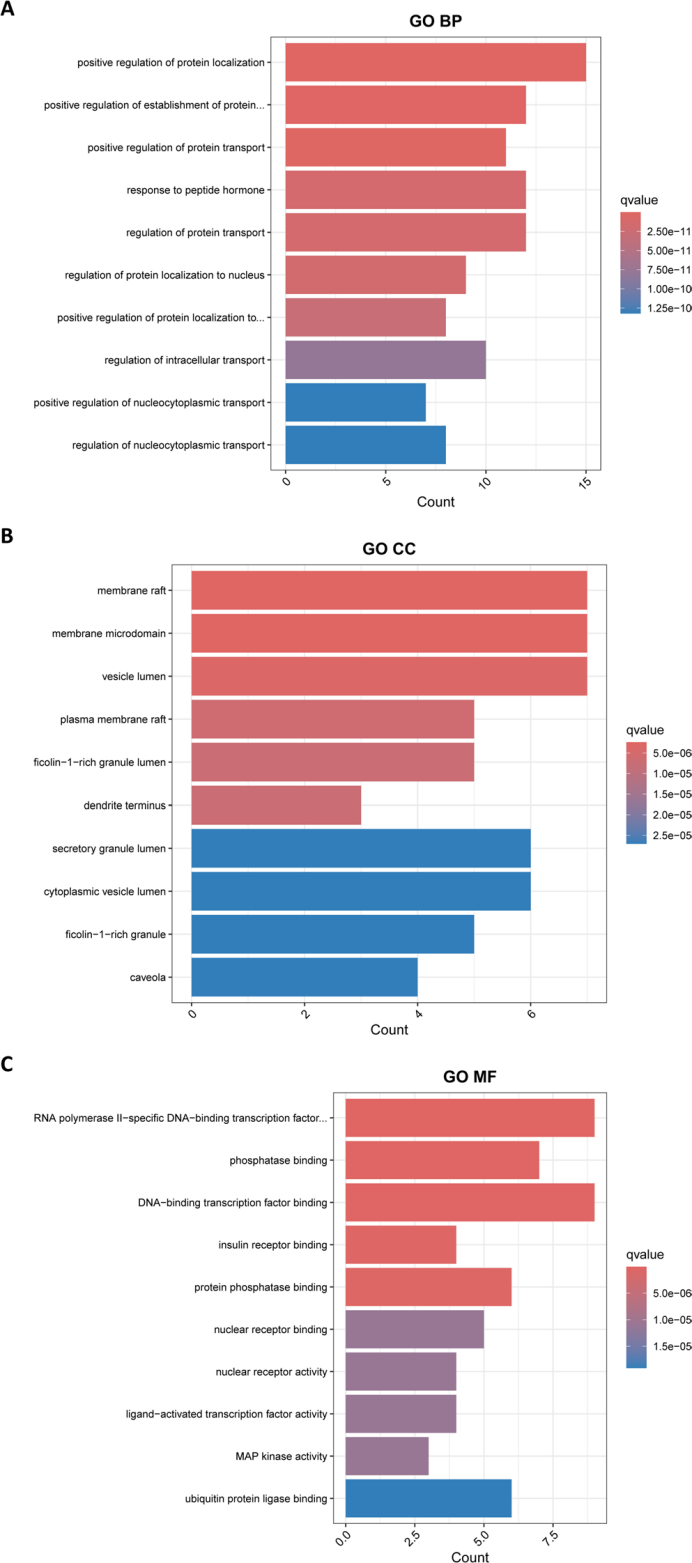
GO enrichment analysis of overlapping targets. GO functional enrichment analysis was performed for the overlapping targets of ligustilide and ALI, including BP, CC, and MF categories. The top enriched GO terms are displayed based on adjusted *P*-values. Bubble size represents gene counts, and color intensity corresponds to statistical significance

GO enrichment analysis of core targets was conducted across three categories: BP, CC, MF. A total of 1,685 statistically significant GO terms were identified, including 1,503 BP, 41 CC, and 140 MF entries with the top 10 enriched terms in each category (ranked by adjusted P-value) visualized in Fig. 4. The targets of ligustilide for treating ALI were predominantly enriched in biological processes, primarily involving the regulation of protein localization and transport—especially nucleocytoplasmic transport—and the response to peptide hormone stimuli, reflecting its core roles in cellular signal transduction, gene expression regulation, and systemic functional integration. Cellular component enrichment centered on membrane rafts and their substructures, as well as vesicle trafficking systems, highlighting its spatial regulatory mechanisms for subcellular signal transduction, neurotransmitter release, and immune responses. Additionally, molecular functions of ligustilide targets included RNA polymerase II-specific DNA-binding transcription factor binding, phosphatase binding, DNA-binding transcription factor binding, and insulin receptor binding, indicating direct modulation of transcriptional and signaling cascades.

#### KEGG pathway enrichment analysis

**Fig. 5:**
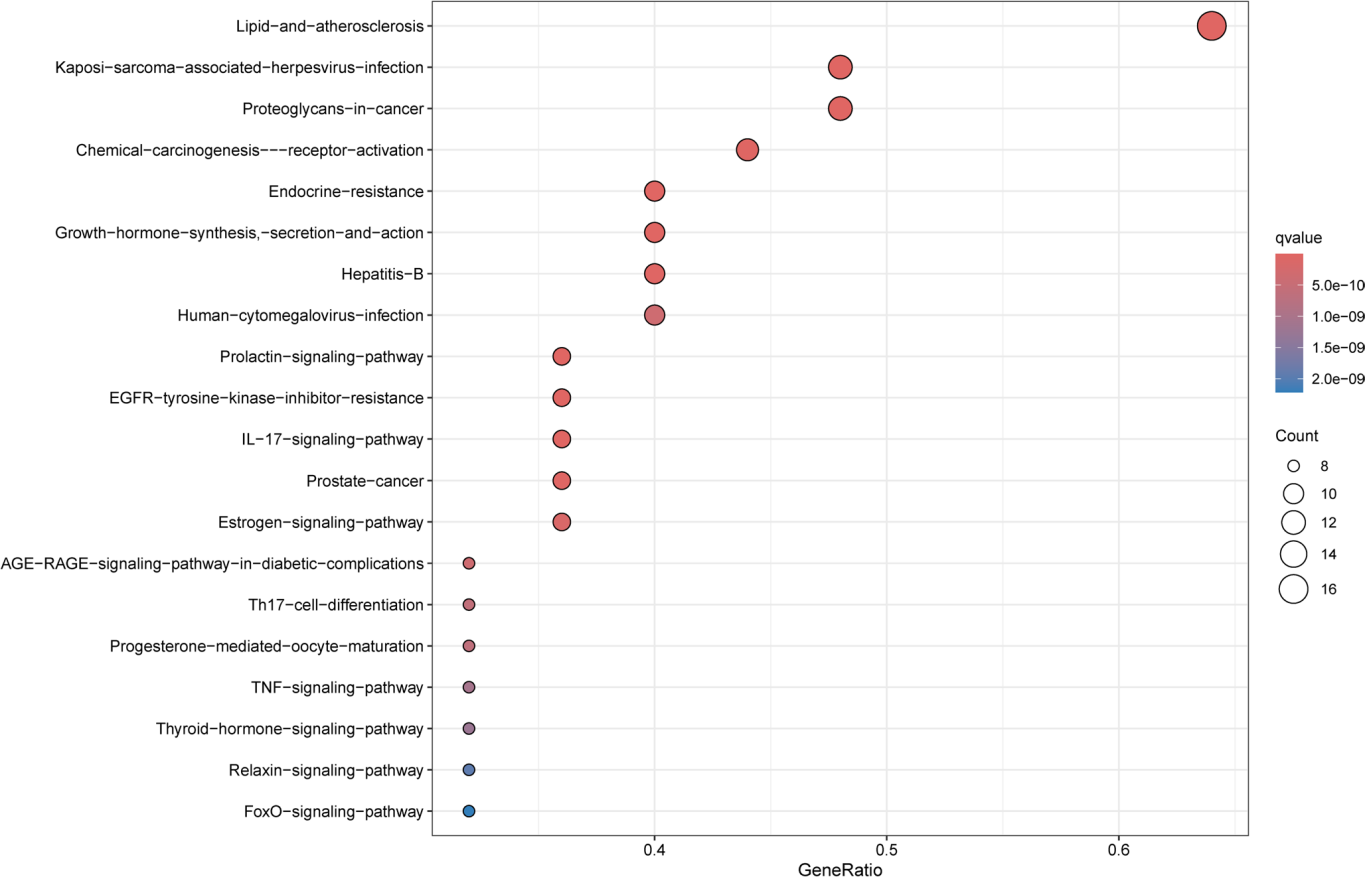
KEGG pathway enrichment analysis of overlapping targets. KEGG pathway enrichment analysis was conducted to identify signaling pathways potentially associated with the therapeutic effects of ligustilide against ALI. The top enriched pathways are displayed based on adjusted *P*-values. Bubble size represents gene counts, and color intensity reflects statistical significance

KEGG pathway analysis via the DAVID database identified 160 enriched pathways, with the top 30 displayed (Fig. 5). A network mapping “active ingredient-disease-signaling pathway-target” was generated using Cytoscape 3.8.0 (Supplementary Figure S3). The top three pathways included Lipid and atherosclerosis, Kaposi sarcoma-associated herpesvirus infection, and Proteoglycans in cancer. Further focus on the PI3KRI-Akt signaling pathway—closely associated with the core target PIK3R1—revealed its pivotal role in ligustilide’s anti-ALI mechanism. These findings suggest that ligustilide exerts anti-ALI effects through multi-pathway interactions, with the PI3K-Akt signaling pathway serving as a central mediator.

#### Molecular docking

**Fig. 6:**
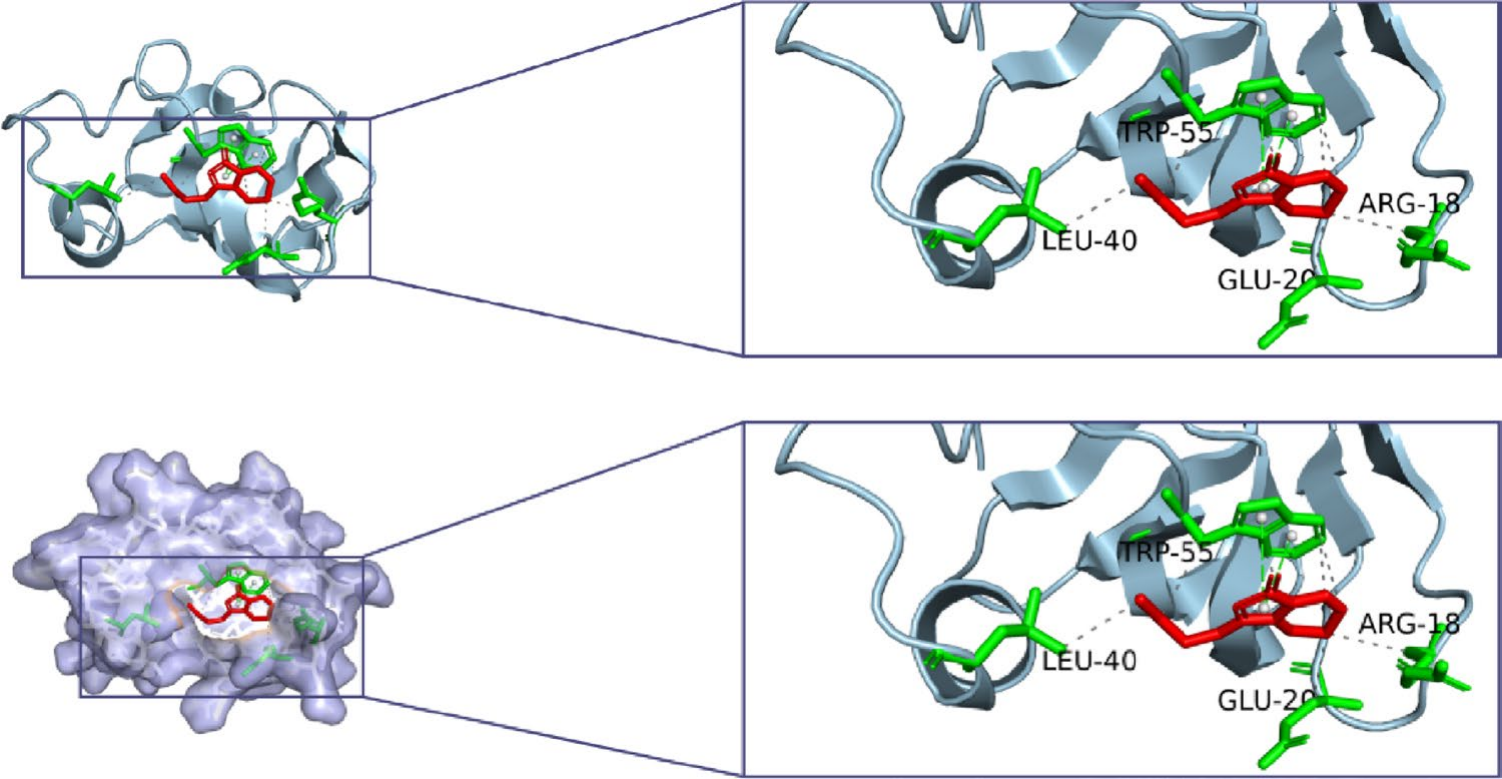
Ligustilide binding to PIK3R1. Molecular docking between ligustilide and PIK3R1 performed using AutoDock Vina; binding conformations visualized in PyMOL

To evaluate the binding affinity between ligustilide and the potential target PIK3R1, molecular docking was performed using AutoDock 1.5.7 software, with visualization processed in pyMOL (Fig. 6). The binding energy between PIK3R1 and ligustilide was − 6.1 kcal/mol, demonstrating a strong binding capability. Analysis of the interacting residues revealed that ligustilide binds within the strongly hydrophobic active pocket of PIK3R1’s catalytic center. This binding pocket is composed of hydrophobic residues LEU-40, TRP-55, GLU-20, and ARG-18, which collectively stabilize ligustilide within the protein’s active site. This result suggests that PIK3R1 may represent a potential binding target of ligustilide in HPAEpiC.

### Cell experiment results

#### Optimal time of action for LPS

**Fig. 7:**
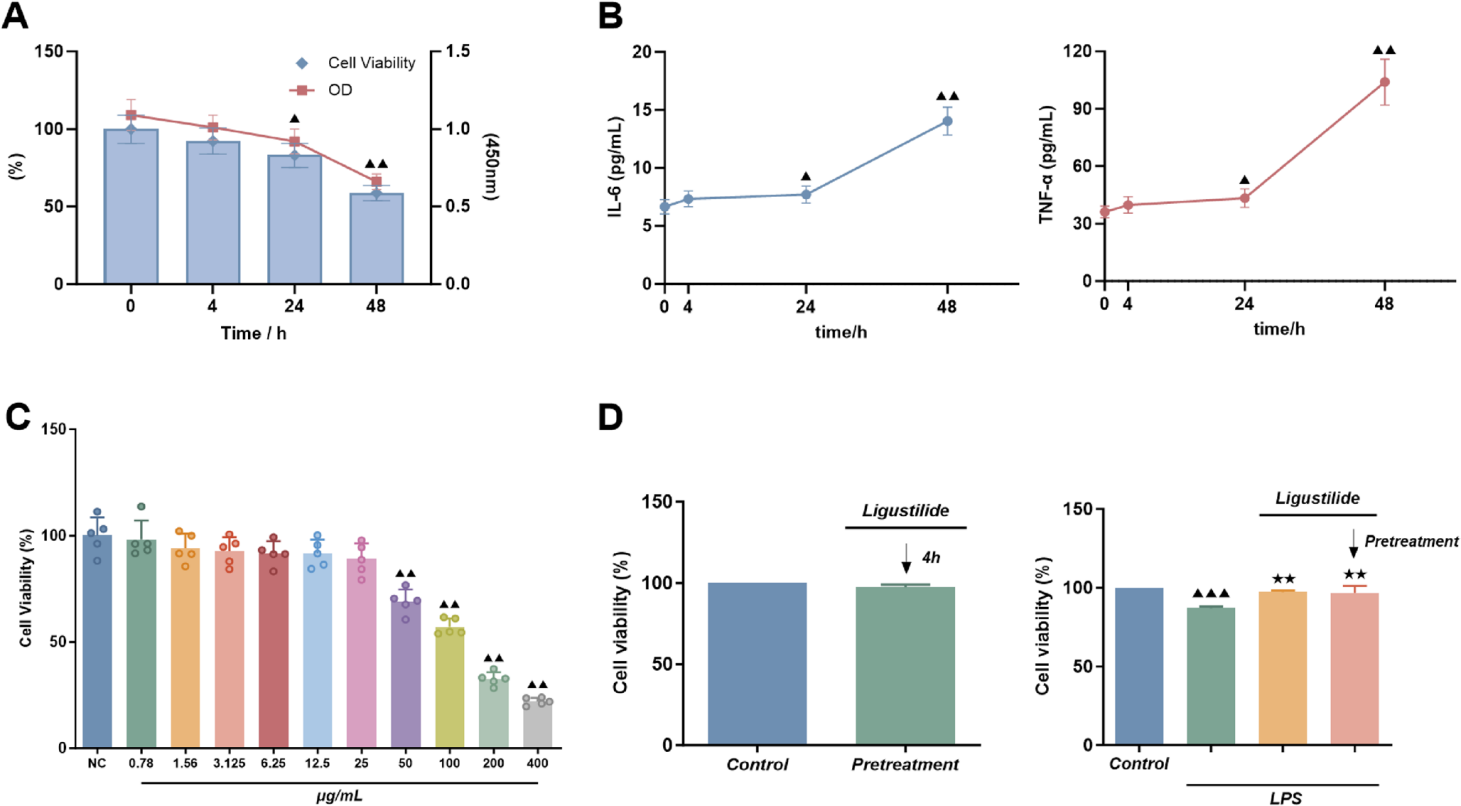
MTT and ELISA experimental results. (**A**) MTT assay showing the effect of 10 µg/ml LPS applied for 0, 4, 24, and 48 h on cell viability (*n* = 5 wells). (**B**) ELISA analysis of IL-6 and TNF-α levels after stimulation with 10 µg/ml LPS for 0, 4, 24, and 48 h (*n* = 6 wells). (**C**) Effect of different concentrations of ligustilide (0.78–500 µg/ml) administered for 24 h on HPAEpiC viability (*n* = 5 wells). (**D**) Cell viability under LPS challenge for 24 h with different ligustilide administration schedules (*n* = 6 wells): Control, LPS (10 µg/ml), simultaneous co-treatment with ligustilide (25 µg/ml) + LPS (10 µg/ml), and ligustilide pretreatment for 4 h (25 µg/ml) followed by LPS exposure (10 µg/ml) with ligustilide maintained during LPS stimulation. Data are expressed as mean ± SD. Statistical analysis for all panels: one-way ANOVA followed by Dunnett’s post hoc test. Compared with 0 h or NC or Control as indicated, ^▲^*P <* 0.05, ^▲▲^*P <* 0.01, ^▲▲▲^*P <* 0.001; compared with LPS group, ^★★^*P <* 0.01

The optimal time of action was determined to be 48 h because, as shown in Fig. 7A, the survival rate of HPAEpiC was significantly decreased (*P <* 0.05 or *P <* 0.01) after 10 µg/ml LPS action for 4 h, 24 h, and 48 h compared to 0 h. This indicates that the cell activity was significantly reduced, and the degree of reduction was positively correlated with the time of action.

#### LPS induces the increased release of inflammatory factors from HPAEpiC

As can be seen in Fig. 7B, the levels of IL-6 and TNF-α in the supernatant of HPAEpiC cultures after 4 h, 24 h and 48 h of action were significantly higher (*P <* 0.05 or *P <* 0.01) compared to 0 h of action of 10 µg/ml LPS.

#### Optimal action concentration of ligustilide

Figure 7C shows that ligustilide concentrations of 0.78, 1.56, 3.125, 6.25, 12.5 and 25 µg/ml had no significant impact on HPAEpiC activity, while concentrations of 50, 100, 200, and 400 µg/ml significantly decreased HPAEpiC activity (*P <* 0.01). As a result, 25 µg/ml was chosen as the ligustilide concentration with the best results.

#### Ligustilide improves cell viability under LPS challenge

As shown in Fig. 7D, compared with the control group, LPS markedly reduced HPAEpiC viability at the experimental endpoint (24 h). Co-treatment with ligustilide significantly improved cell viability relative to the LPS group, indicating a protective effect of ligustilide against LPS-induced injury. Notably, no obvious difference in endpoint viability was observed between the 4 h pretreatment schedule and the simultaneous co-treatment schedule, suggesting that the cytoprotective effect of ligustilide is relatively robust with respect to administration timing in this viability readout.

#### Ligustilide and anti-IL-17 A neutralizing antibodies attenuate LPS-induced inflammatory responses in HPAEpiC

**Fig. 8:**
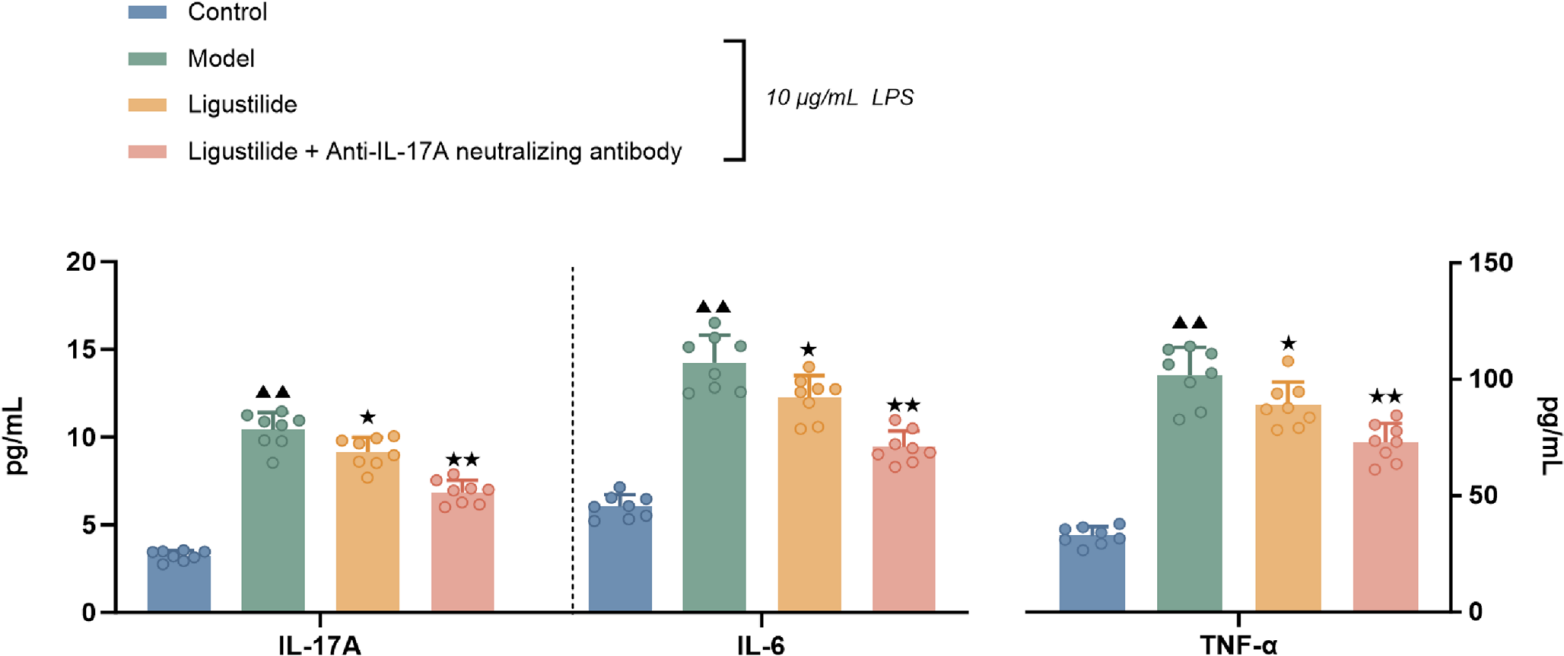
Levels of IL-17 A, IL-6 and TNF-α in the supernatant of HPAEpiC cultures in each group. Cells were pretreated for 4 h with ligustilide (25 µg/ml) alone or in combination with anti-IL-17 A neutralizing antibody (1:500), followed by stimulation with LPS (10 µg/ml) for 24 h. IL-17 A, IL-6 and TNF-α levels in the culture supernatants were determined by ELISA. Data are presented as mean ± SD (*n* = 8 wells). Groups separated by dotted lines indicate independent experimental sets. Statistical analysis was performed using one-way ANOVA followed by Dunnett’s post hoc test. Compared with normal control group, ^▲▲^*P <* 0.01; compared with LPS model group, ^★^*P <* 0.05, ^★★^*P <* 0.01

As shown in Fig. 8, the concentrations of IL-17 A, IL-6, and TNF-α in the supernatant of HPAEpiC cultures were considerably higher in the LPS model group than in the normal control group (*P <* 0.01). In contrast, the concentration levels of IL-17 A, IL-6, and TNF-α were significantly lower in the ligustilide group compared to the LPS model group, the levels of these inflammatory factors were further reduced in the ligustilide+anti-IL-17 A neutralizing antibody group (*P <* 0.05 or *P <* 0.01).

#### Ligustilide and anti-IL-17 A neutralizing antibodies attenuate LPS-induced autophagy deficiency in HPAEpiC

##### Determination of mRNA expression levels of Beclin-1 and LC3B in cells by real Time-PCR

**Fig. 9:**
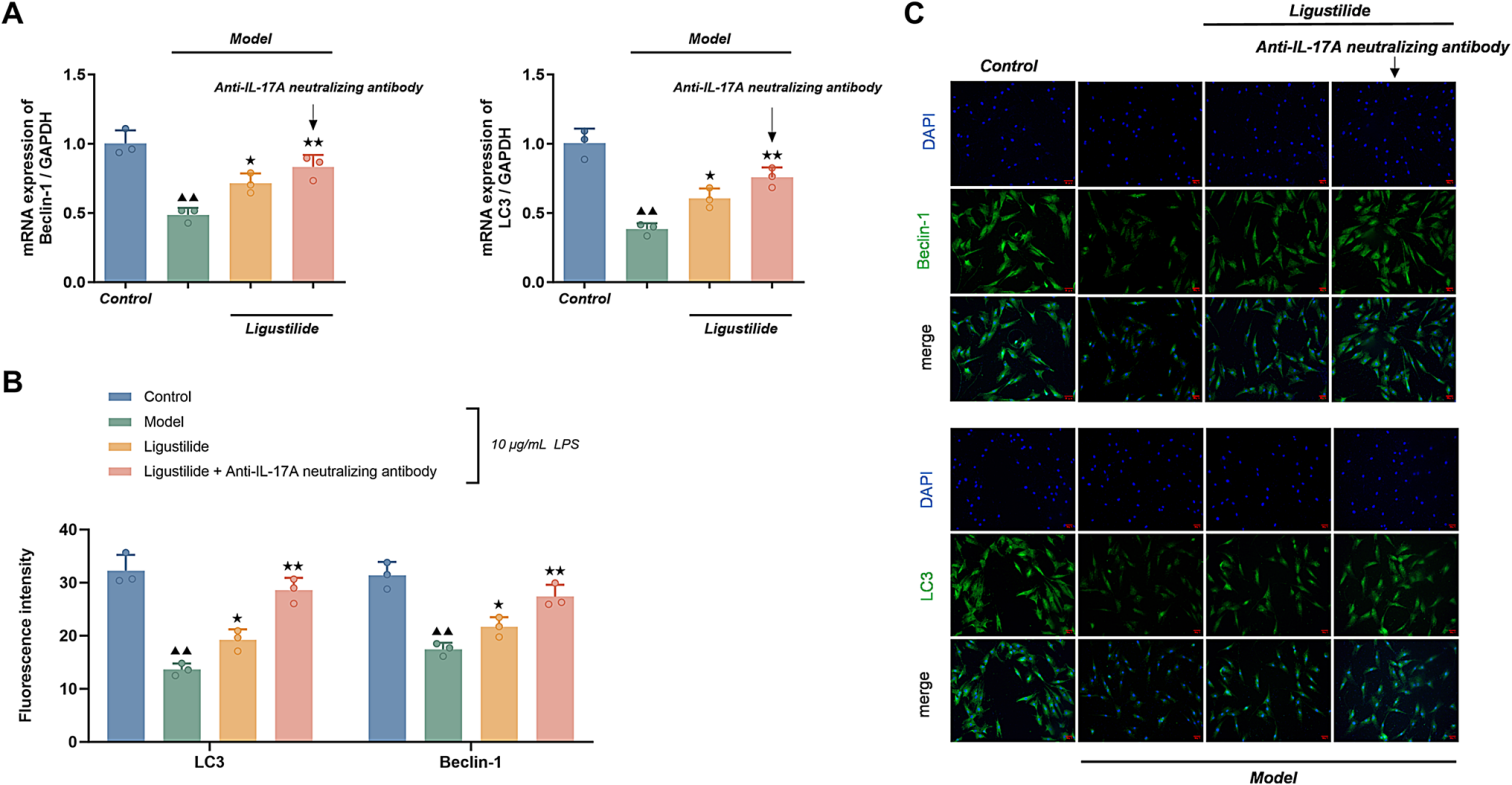
Ligustilide and anti-IL-17 A neutralizing antibodies attenuate LPS-induced autophagy deficiency in HPAEpiC. (**A**) mRNA expression of Beclin-1 and LC3 in HPAEpiC. HPAEpiC were pretreated for 4 h with ligustilide (25 µg/ml) alone or in combination with anti-IL-17 A neutralizing antibody (1: 500), and then exposed to LPS (10 µg/ml) for 24 h in the continued presence of ligustilide±antibody. Beclin-1 and LC3 mRNA levels were quantified by real-time PCR. Data are expressed as mean ± SD (*n* = 3 biological replicates). (**B**) Quantification of Beclin-1 and LC3 immunofluorescence under the same treatment conditions. Data are expressed as mean ± SD (*n* = 3 independent experiments). (**C**) Representative immunofluorescence images of Beclin-1 and LC3 in each group (multiplicity: 200×). All images were obtained using identical acquisition parameters. Scale bar variations reflect figure layout adjustments rather than differences in magnification. Control group: scale bar = 50 μm. Other group: scale bar = 100 μm. Statistical analysis was performed using one-way ANOVA followed by Dunnett’s post hoc test. Compared with normal control group, ^▲▲^*P <* 0.01; compared with LPS model group, ^★^*P <* 0.05, ^★★^*P <* 0.01

(Fig. 9A) The expression levels of Beclin-1 and LC3B mRNA in HPAEpiC of the LPS model group were lower compared to the normal control group (*P <* 0.01); the expression levels of Beclin-1 and LC3B mRNA in HPAEpiC of the ligustilide group were significantly higher compared to the LPS model group, and the expression levels of ligustilide + anti-IL-17 A The expression levels of Beclin-1 and LC3B mRNA were substantially higher in the ligustilide + anti-IL-17 A neutralizing antibody group (*P <* 0.05 or *P <* 0.01).

##### Detection of cellular Beclin-1 and LC3 staining intensity by Immunofluorescence

Figure 9B and C illustrates that the staining intensity of Beclin-1 and LC3 in HPAEpiC of the LPS model group were markedly smaller than those of the normal control group (*P <* 0.01); compared with the LPS model group, the staining intensity of Beclin-1 and LC3 in HPAEpiC of the ligustilide group were significantly higher, and the staining intensity of the ligustilide + anti-IL-17 A neutralizing antibody group were further increased (*P <* 0.05 or *P <* 0.01).

##### Autophagy of HPAEpiC in each group observed by transmission electron microscopy

**Fig. 10:**
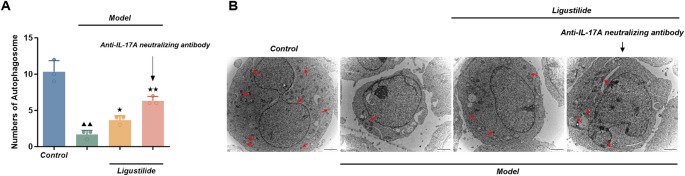
Ultrastructural autophagy changes observed by transmission electron microscopy in HPAEpiC. (**A**) Quantification of autophagic vesicles. HPAEpiC were pretreated for 4 h with ligustilide (25 µg/ml) alone or together with anti-IL-17 A neutralizing antibody (1:500), followed by LPS (10 µg/ml) stimulation for 24 h in the continued presence of ligustilide±antibody. The number of autophagic vesicles per cell was quantified from TEM images. Data are presented as mean ± SD (*n* = 3 biological replicates). Statistical analysis was performed using one-way ANOVA followed by Dunnett’s post hoc test. Compared with normal control group, ^▲▲^*P <* 0.01; compared with LPS model group, ^★^*P <* 0.05, ^★★^*P <* 0.01. (**B**) Representative TEM images of autophagosomes in each group (multiples: 10,000×). Red arrows indicate autophagosomes

The number of autophagic vesicles in HPAEpiC of the model group was significantly decreased (*P <* 0.01) compared to the normal control group, and the depth of cellular autophagy was reduced. In contrast, the number of autophagic vesicles in HPAEpiC of the ligustilide and ligustilide+anti-IL-17 A neutralizing antibody groups was significantly increased (*P <* 0.05 or *P <* 0.01) compared to the model group (Fig. 10A). In particular, cellular autophagy deepened and the number of autophagic vesicles increased after the combination of anti-IL-17 A neutralizing antibodies (Fig. 10B).

#### Ligustilide and anti-IL-17 A neutralizing antibodies attenuate the activation of the PI3K/Akt/mTOR signaling pathway by LPS

**Fig. 11:**
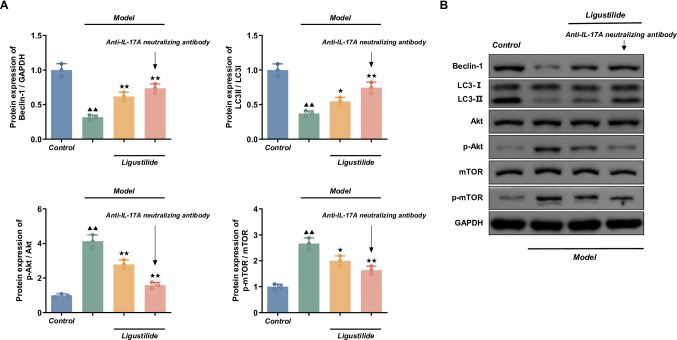
Ligustilide and anti-IL-17 A neutralizing antibodies attenuate the activation of the PI3K/Akt/mTOR signaling pathway by LPS. (**A**) Quantification of Beclin-1, LC3-Ⅱ, p-Akt/Akt and p-mTOR/mTOR protein expression. Cells were pretreated for 4 h with ligustilide (25 µg/ml) alone or in combination with anti-IL-17 A neutralizing antibody (1:500), and then stimulated with LPS (10 µg/ml) for 24 h in the continued presence of ligustilide±antibody. Protein levels were analyzed by Western blot and normalized to GAPDH. Data are expressed as mean ± SD (*n* = 3 biological replicates). Statistical analysis was performed using one-way ANOVA followed by Dunnett’s post hoc test. Compared with normal control group, ^▲^*P <* 0.05, ^▲▲^*P <* 0.01; compared with LPS model group, ^★^*P <* 0.05, ^★★^*P <* 0.01. (**B**) Representative immunoblots of Beclin-1, LC3-Ⅱ, Akt, p-Akt, mTOR and p-mTOR in each group

As shown in Fig. 11, compared with the normal control group, the expression levels of Beclin-1 and LC3-Ⅱ band intensity were considerably reduced (*P <* 0.01) and the expression levels of p-Akt/Akt and p-mTOR/mTOR protein were significantly higher (*P <* 0.01) in the HPAEpiC of the LPS model group.

Beclin-1 and LC3-Ⅱ protein expression levels in the ligustilide group and the ligustilide+anti-IL-17 A group were significantly higher (*P <* 0.05 or *P <* 0.01) and the expression levels of p-Akt/Akt and p-mTOR/mTOR protein were lower (*P <* 0.05 or *P <* 0.01) when compared to the LPS model group. The group treated with ligustilide and an anti-IL-17 A neutralizing antibody was more different.

## Discussions

In-depth study of the pathogenesis of ALI and early interruption of the pathogenesis, especially the death of lung parenchymal cells, is key to slowing disease progression and reducing the mortality rate.

In pathological states, the activation or inhibition of autophagy, which is regulated by multiple signalling pathways, has protective and detrimental effects on disease development [23–25]. When cells are damaged, autophagy serves as a defense mechanism to remove damaged organelles and metabolites from the cytoplasm, remodeling the subcellular level and protecting damaged cells. The initiating step of autophagy activation involves the formation of phagocytic vesicles by the precursor membranes of autophagic vesicles. This encapsulates cytoplasmic material, which, when attacked by lipopolysaccharide in the cell wall and when bound to TLR4 (Toll-like receptor 4) [26], inhibits mTOR activity on the surface of lysosomes. The mammalian homolog of ULK1 (unc-51-like kinase 1) and ATG 13 (autophagy-related gene 13) are then rapidly dephosphorylated, inducing autophagy [27]. Vps34 is a class phosphatidylinositol-3-kinases (PIK3C3) in mammals. Phagocytic vesicles activate and promote Vps34 binding to the homolog of ATG 6 Beclin-1. Together, they nucleate membrane vesicles promoting the localization of autophagy proteins to autophagic vesicles for further stretching and expansion [28–30].

**Fig. 12:**
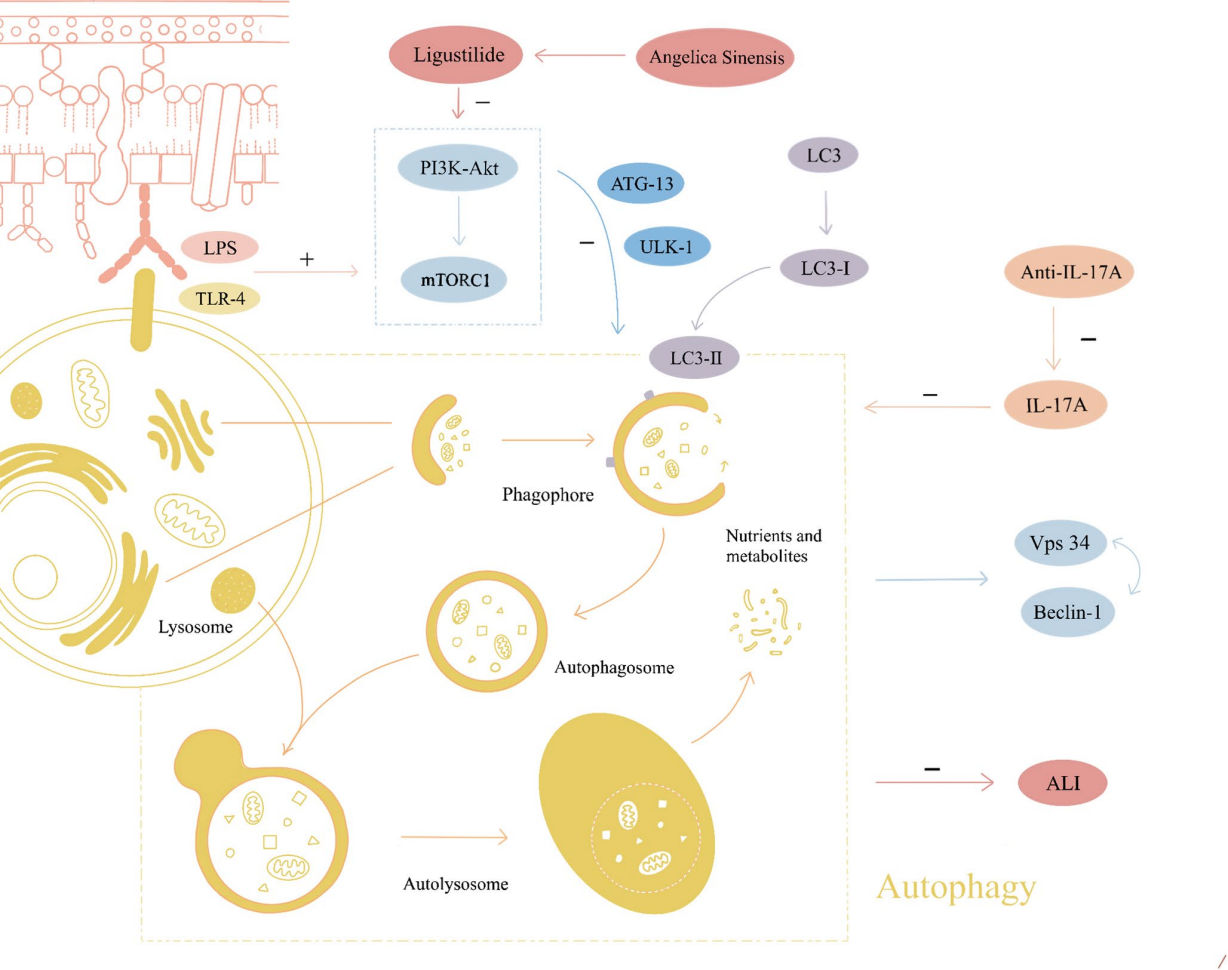
The putative mechanism and signaling pathway are thought to be that ligustilide modulates autophagy-related pathways in LPS-induced ALI HPAEpiC

In addition, microtubule-associated protein 1 light chain 3 (MAP1LC3), which targets the autophagosomal membrane, is involved in the extension phase of autophagy and consists of two interconvertible forms; the lysis form (LC3-Ⅰ) and the lipolysis form (LC3-Ⅱ). When autophagy occurs, LC3-Ⅰ is modified by ubiquitin-like processing and binds to phosphatidylethanolamine to form LC3-Ⅱ. LC3-Ⅱ is always located on the autophagic vesicle membrane and is considered an autophagosome marker molecule that increases with autophagic membranes [31] (Fig. 12).

Alveolar epithelial cells play an important role in maintaining the stability and structure of the internal alveolar environment. They are the main damaged target cells in the pathogenesis of ALI. Previous studies have been reported that LPS stimulation inhibits autophagy in alveolar epithelial cells through mTOR activation, thereby exacerbating lung injury. Conversely, suppression of mTOR signaling restores autophagic activity and alleviates LPS-induced ALI [26, 32]. This suggests that insufficient autophagy is one of the pathogenic mechanisms of LPS-induced ALI, whereas appropriate activation of autophagy confers protective effects on lung tissue [33].

PI3K/Akt targets mTOR, mediating various physiological functions, including cell proliferation, differentiation, migration, and apoptosis [34], and represents a central regulatory axis in autophagy. During normal physiological processes, growth factors stimulate the PI3K/Akt pathway and activate the mTOR complex mTORC1 through phosphorylation. This, in turn, negatively regulates a complex containing molecules such as the autophagy-associated proteins ATG13 and ULK1. Studies have confirmed that the PI3K/Akt/mTOR signaling pathway is a key inhibitory signaling pathway for autophagy.

In summary, appropriate inhibition of the PI3K/Akt/mTOR signaling pathway induces enhanced autophagic activity in lung epithelial cells. It is gradually being recognized as a potential therapeutic strategy for ALI.

Current therapeutic approaches for ALI are mainly supportive and have substantial limitations, including hyperventilation, hepatic and renal toxicity, and bone marrow suppression. Traditional Chinese medicine has attracted increasing attention for ALI treatment due to its multi-component, multi-target characteristics and relatively low toxicity. Such multi-target pharmacological features may be particularly advantageous in complex inflammatory diseases like ALI, where multiple signaling pathways, including inflammatory and autophagy-related networks, are simultaneously dysregulated.

Notably, activation of this pathway is tightly regulated by upstream inflammatory signals. In the context of LPS-induced lung injury, Toll-like receptor 4 (TLR4) serves as a primary pattern recognition receptor that initiates downstream signaling cascades, including NF-κB activation, leading to excessive production of pro-inflammatory cytokines. Accumulating evidence suggests that TLR4/NF-κB signaling can intersect with the PI3K/Akt/mTOR pathway, thereby modulating autophagic activity and inflammatory responses in epithelial cells [35]– [36]. Such pathway crosstalk further provides indirect support for the potential therapeutic efficacy of traditional Chinese medicine, which is characterized by a multi-target mode of action.

Our previous studies showed that *Angelica sinensis* exert potential protective effects against acute lung injury (ALI). In particular, its compound formulation reduced the secretion of inflammatory factors such as IL-17, TNF-α, and IL-6 through the TLR4/caveolin-1 pathway, thereby alleviating alveolar structural damage and improving ALI [37]. However, the instability of compound prescriptions and the diversity of a single herb limited our research on specific drug targets and the detailed protective mechanisms of ALI. Therefore, we conducted a detailed analysis of ligustilide, the main active ingredient of *Angelica sinensis*. Our results suggested that ligustilide effectively reduced inflammatory factors such as IL-17 A, IL-6, and TNF-α in lung tissue and improved ALI [38]. The present study further confirms these results.

It should be noted that the concentrations of ligustilide used in the present in vitro experiments are higher than plasma concentrations typically achievable under clinical conditions. This discrepancy is commonly observed in cell-based studies, as the absence of metabolic clearance, protein binding, and tissue distribution often necessitates higher compound concentrations to elicit measurable biological effects.

From a pharmacokinetic perspective, previous studies have reported that Z-ligustilide exhibits favorable lipophilicity and membrane permeability; however, its oral bioavailability is relatively low due to extensive first-pass metabolism [39]. In addition, preclinical pharmacokinetic studies indicate rapid systemic clearance of ligustilide, resulting in limited plasma exposure following oral administration [40].

However, these pharmacokinetic characteristics do not necessarily preclude biological efficacy, particularly in the context of traditional herbal medicine. In clinical practice, *Angelica sinensis* is administered as a multi-component formulation, in which ligustilide represents one of several bioactive constituents that may exert synergistic or complementary effects. Moreover, transient exposure and rapid tissue distribution of ligustilide may be sufficient to modulate early inflammatory signaling events, such as TLR4-associated pathways and autophagy-related processes, which play critical roles during the initiation phase of acute lung injury. Therefore, the present in vitro findings primarily provide mechanistic insight rather than direct evidence for optimal clinical dosing.

A closer look at our experimental design may also help explain why the 4 h pretreatment and the non-pretreatment schedules did not differ significantly in endpoint viability. One possibility is that ligustilide initiates its protective actions relatively quickly, so extending exposure by an additional 4 h does not translate into a measurable gain at the final time point. Another possibility is that MTT-based viability is an overall, integrative readout; any timing-dependent differences may be more evident in earlier mechanistic indicators, such as upstream signaling activity, cytokine production, or autophagy-related markers.

Interleukin 17 A (IL-17 A) is an important pro-inflammatory cytokine that induces the expression and release of TNF-α, IL-6, and other inflammatory factors and chemokines, causing inflammatory cell infiltration and tissue destruction, exacerbating the inflammatory response. In a mouse model of LPS-induced ALI, intervention with IL-17 knockout or IL-17Ab can effectively alleviate ALI, so IL-17 A also has an important pathogenic role in ALI caused by LPS [41]. In another study, after blocking IL-17 A function with inhibitors, lung histopathological damage in mechanically ventilated mice was significantly improved, with further upregulation of Beclin-1 and LC3-Ⅱ expression and enhanced autophagy, demonstrating that IL-17 A may be involved in the pathophysiological process of lung injury associated with mechanical ventilation by inhibiting autophagic activity. This study also found that the ratios of p-PI3K/PI3K, p-Akt/Akt, and p-mTOR/mTOR were decreased, and autophagy was further enhanced after blocking IL-17 A. This suggests that IL-17 A may regulate autophagic activity in mechanical ventilation-related lung injury by activating the PI3K/Akt/mTOR signaling pathway [42]. However, unlike this pathogenic factor, it has not been reported whether IL-17 A modulates autophagic activity by interfering with the PI3K/Akt/mTOR signaling pathway in LPS-induced ALI. Consistent with these findings, our data support the notion that IL-17 A signaling may interact with the PI3K/Akt/mTOR pathway to modulate autophagic activity in LPS-induced epithelial injury, and that combined targeting of IL-17 A and PI3K/Akt/mTOR signaling could exert synergistic protective effects.

Nevertheless, several limitations should be acknowledged. First, this study was conducted exclusively in vitro using HPAEpiCs, which cannot fully recapitulate the complex immune microenvironment of ALI in vivo. Second, the pharmacokinetic properties of ligustilide, including bioavailability, metabolic stability, tissue distribution, and potential toxicity, were not evaluated. Therefore, future studies employing animal models of ALI, together with pharmacokinetic and dose–response analyses, are warranted to further assess the translational potential of ligustilide.  

## Conclusions

In conclusion, the present study demonstrates that ligustilide alleviates LPS-induced autophagic impairment and inflammatory responses in HPAEpiCs. These effects are associated with modulation of the PI3K/Akt/mTOR signaling pathway. Moreover, combined intervention with an IL-17 A-neutralizing antibody further enhanced autophagic activity, suggesting a potential synergistic interaction between IL-17 A signaling and the PI3K/Akt/mTOR pathway. Collectively, these findings provide mechanistic insight into the protective effects of ligustilide against LPS-induced epithelial injury and support its potential as a candidate for further investigation in ALI.

## Supplementary Information

Below is the link to the electronic supplementary material.

Supplementary Material 1

Supplementary Material 2

Supplementary Material 3

Supplementary Material 4

Supplementary Material 5

Supplementary Material 6

Supplementary Material 7

Supplementary Material 8

Supplementary Material 9

Supplementary Material 10

Supplementary Material 11

## Abbreviations

LPS: lipopolysaccharide
ALI: acute lung injury
PI3K: phosphatidylinositol-3 kinase
Akt: protein kinase B
mTOR: mammalian target of rapamycin
IL-17A: interleukin-17A
HPAEpiC: human pulmonary alveolar epithelial cells
IL-6: interleukin-6
TNF-α: tumour necrosis factor-alpha
Beclin-1: coiled-coil, myosin-like BCL2 interacting protein
LC3B: microtubule-associated protein 1 light chain 3 beta, MAP1LC3B
LC3-Ⅱ: microtubule-associated protein 1 light chain 3, lipolytic forms
p-mTOR: phospho-Mammalian target of rapamycin
p-Akt: phospho-protein kinase B
