# Supplementary figures and images for: A novel mechanism for treating acute lung injury with ligustilide: elucidation via network pharmacology and in vitro validation
Source: J Nat Med. 2026 Mar 21;80(3):639–56. doi: 10.1007/s11418-026-02011-y (PMC13186842; doi:10.1007/s11418-026-02011-y)

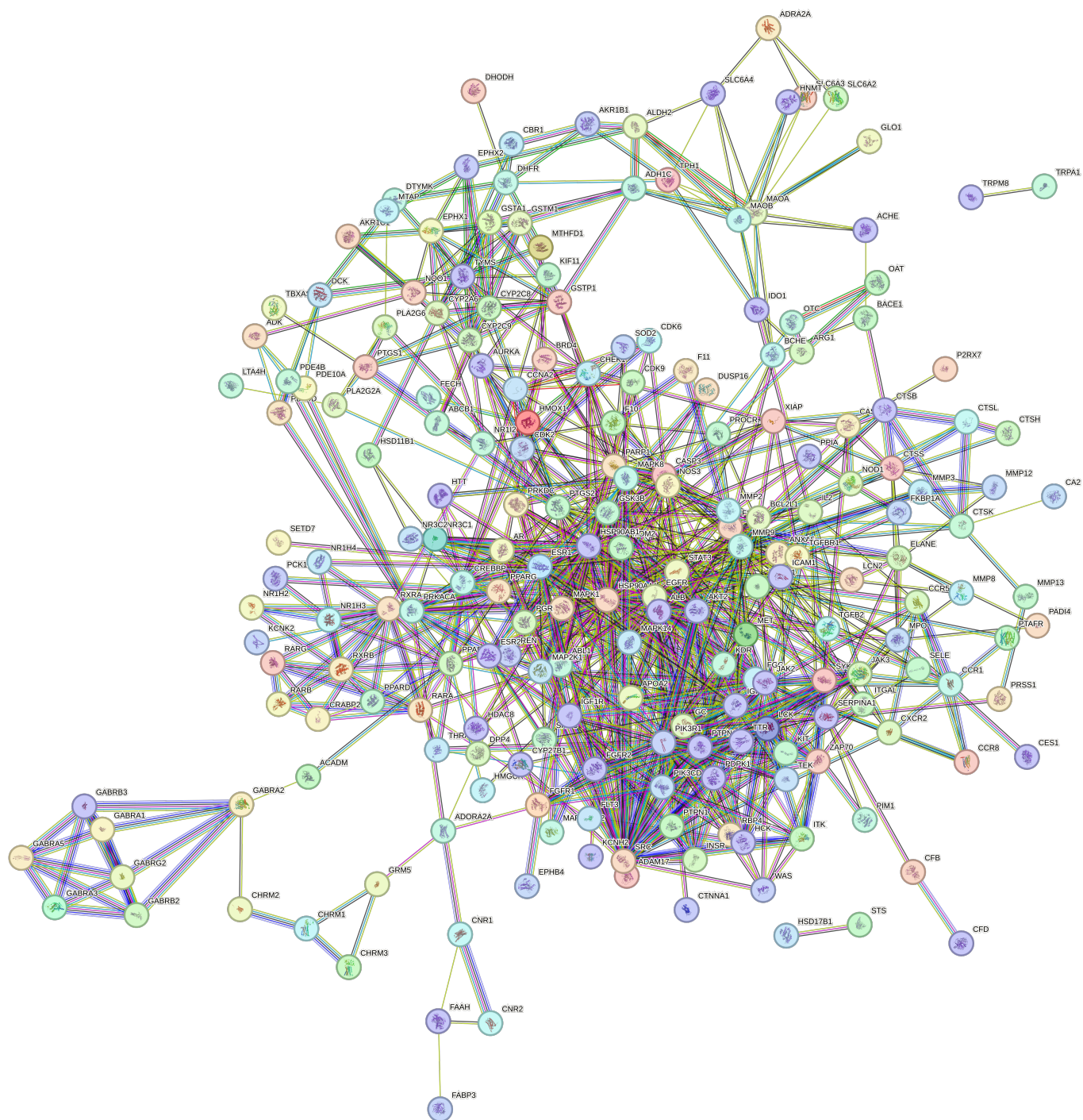

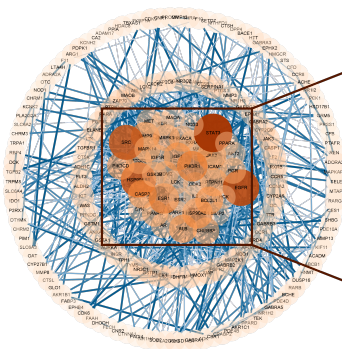

211Nodes 865edgse

$DC > 12$

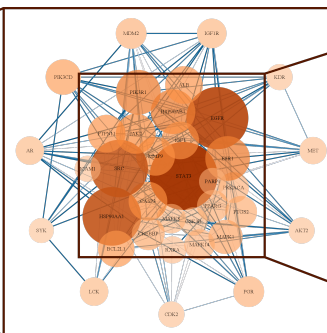

36Nodes 238edgse

$BC > 273$

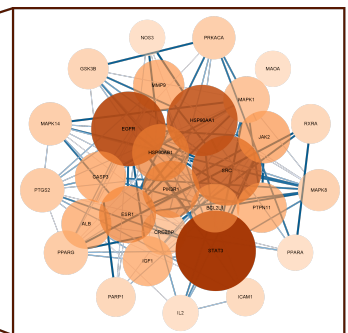

25Nodes 129edgse

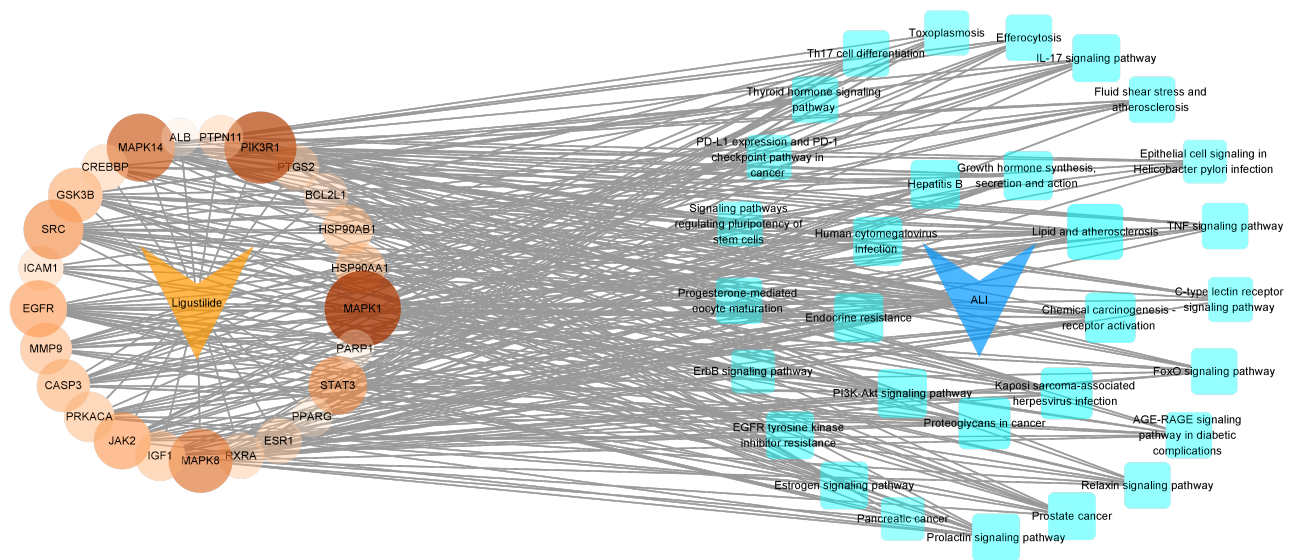

Supplement: Supplementary file 10 — Supplementary Material 10 [file 11418_2026_2011_MOESM10_ESM.pdf]
