# Supplementary material for: A novel mechanism for treating acute lung injury with ligustilide: elucidation via network pharmacology and in vitro validation
Source: J Nat Med. 2026 Mar 21;80(3):639–56. doi: 10.1007/s11418-026-02011-y (PMC13186842; doi:10.1007/s11418-026-02011-y)
Supplement: Supplementary file 11 — Supplementary Material 11 [file 11418_2026_2011_MOESM11_ESM.docx]

**Figure S1.** PPI network of overlapping targets.

The 227 overlapping targets between ligustilide and ALI were imported into the STRING database (species: Homo sapiens; minimum interaction score ≥ 0.70). Disconnected nodes were hidden. The network was visualized using Cytoscape 3.8.0. Nodes represent proteins, and edges represent protein–protein associations.

**Figure S2.** Core target network derived from topological analysis.

Core targets were identified using the CytoNCA plugin in Cytoscape based on Degree Centrality (DC) and Betweenness Centrality (BC) values exceeding twice the median thresholds. Nodes represent core proteins potentially involved in the therapeutic effects of ligustilide against ALI.

**Figure S3.** “Compound-disease-pathway-target” interaction network.

The integrative network was constructed based on ligustilide, ALI, enriched KEGG pathways, and overlapping targets. The network was visualized using Cytoscape 3.8.0. Nodes represent compounds, disease entities, signaling pathways, and target proteins. Edges represent predicted or enriched associations.
